# Winning a noble race

**DOI:** 10.1073/pnas.2321322120

**Published:** 2023-12-21

**Authors:** Marcia McNutt

There are some competitions decidedly worth winning. One of the noblest is the competition to be the nation with the highest quality science and innovation, given the critical role of R&D to public health, quality of life, national security, and economic competitiveness. America has for many decades prided itself on the pre-eminence of its science enterprise. However, confidence that American science will continue to lead the world, and even the certainty that it currently does, is waning (1). This perception is not based on opinion only. For example, trends show that China is poised to surpass, or has already passed, the United States in important measures such as innovation, papers in top journals, and science funding (2). It is useful to consider what factors produced and maintained the world-leading quality of US science and what might be contributing to the recent concerns that we are entering a period of decline.

The recent Nobel Prize Symposium (3) hosted by the House of Sweden in Washington, DC, to celebrate the 2023 laureates from the United States prompted me to examine the question: “Why has the US been the one nation that annually can expect to celebrate several Nobel laureates in sciences?” In fact, since the inception of the Nobel prizes in Physics, Chemistry, and Physiology/Medicine in 1901, followed by the Economics^*^ prize in 1969, the United States has garnered 368 prizes (excluding the categories of Peace and Literature), while the nation with the second-highest tally, the United Kingdom, has been awarded 112. Germany is closely behind with 100. In fact, over the past ~75 years since the end of World War II, there have been two or more US Nobel laureates in 65 of those years—an amazing record.

But US dominance was not always the case. Prior to the end of the WWII, Germany led the world with 39 science Nobel Prizes, and again, the United Kingdom was second with 23. The United States could only boast 17 science medals. What changed after WWII that caused the dominance of the United States on the Nobel stage? I compared the US’s Nobel record against three possible explanations for the sudden rise in US competitiveness for the science community’s highest honor: 1) the establishment of a new Nobel in Economic Sciences in 1969; 2) the publication of Vannevar Bush’s *Science, the Endless Frontier* (4), followed on immediately with the founding of the NSF to fund basic research; and 3) the impact of immigration on the competitiveness of US science.

Hypothesis 1, the addition of the Nobel prize in Economic Sciences, would be expected to have an appreciable impact on the US tally of medals because in this one field alone, the United States has been awarded 62 Nobel Prizes in Economics since its inception in 1969. The United Kingdom has the second largest tally in Economics at nine medals, and only 10 nations other than the United States have more than one. However, if we compare the annual number of US medals in science (Fig. 1), it is apparent that while the addition of the Economics prize added to the US tally since 1969, it does not alter the fact that the jump in US Nobels began well before 1969. In support of Hypothesis 2, the number of US Nobels began ramping up soon after *Science: The Endless Frontier,* suggesting that post-war investment in basic research was soon followed by Nobel prizes. The addition of the prizes in Economics merely created an additional step up in the mid-1970s.

**Fig. 1.**
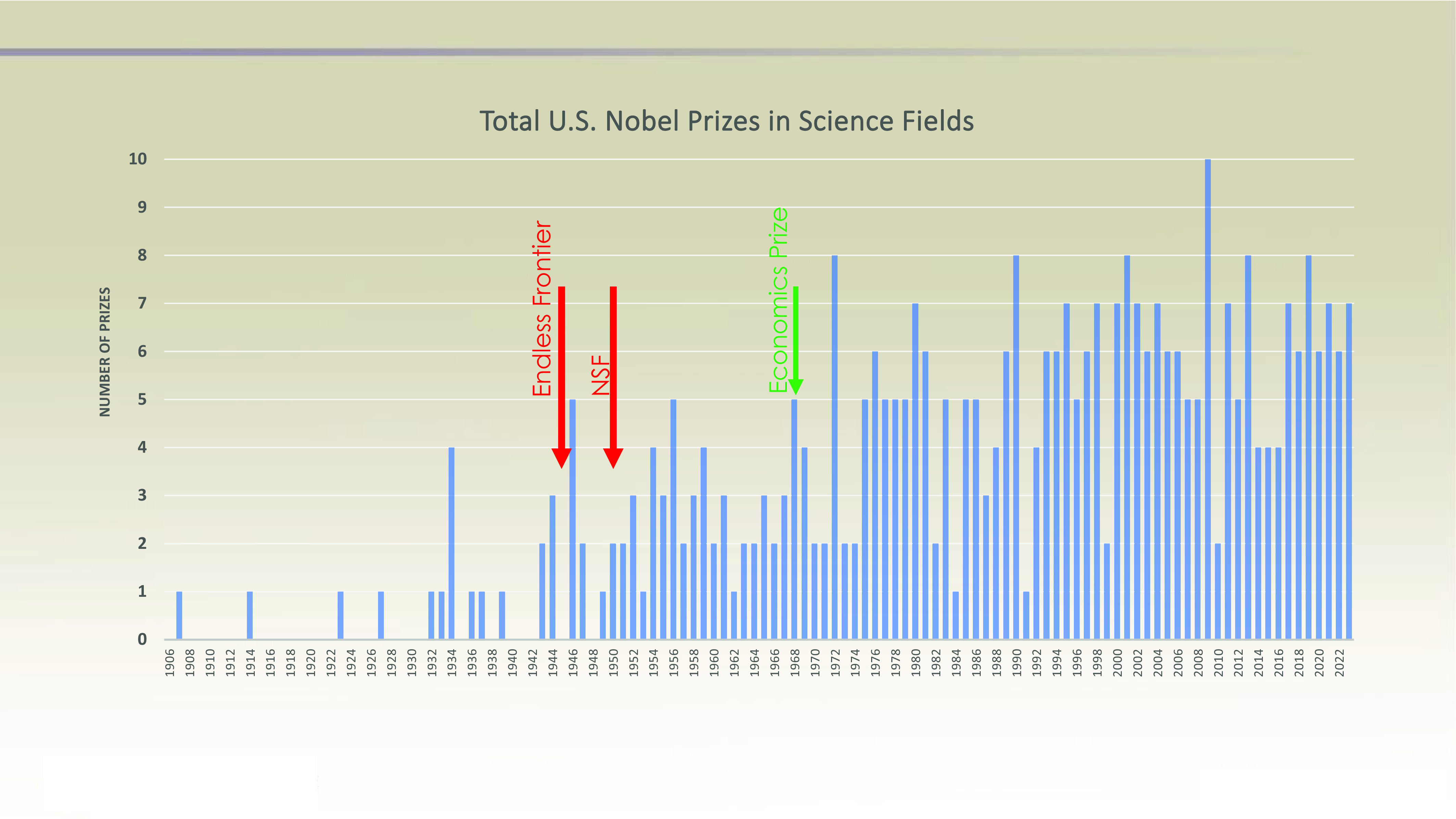
Vertical bars indicate the number of Nobel Prizes for the United States as a function of year in the categories of Physics, Chemistry, Physiology or Medicine, and Economics. Colored arrows indicate the timing of important events: the publication of *Science: The Endless Frontier*, the establishment of the NSF, and the inauguration of the Nobel Prize in Economic Sciences. See Dataset S1.

Perhaps a more interesting association is the connection between Nobel prizes and immigration, Hypothesis 3. In this part of the analysis, I counted the percentage of medals that were earned by citizens of the top 10 nations (in terms of total Nobel prizes in the sciences) who immigrated to that nation after birth, and then plotted each nation’s ranking in terms of total science medals versus the ranking of percentage of those medals that went to immigrants (Fig. 2). For example, in this analysis, the United States ranks #1 in total medals and #1 in terms of the fraction of medals awarded to first-generation immigrants (ranging from a high of 33 percent for Physics to as low as 21 percent for Economics). Russia ranks 10th in the medal count and 10th in the percentage of immigrants (none) in that tally.

**Fig. 2.**
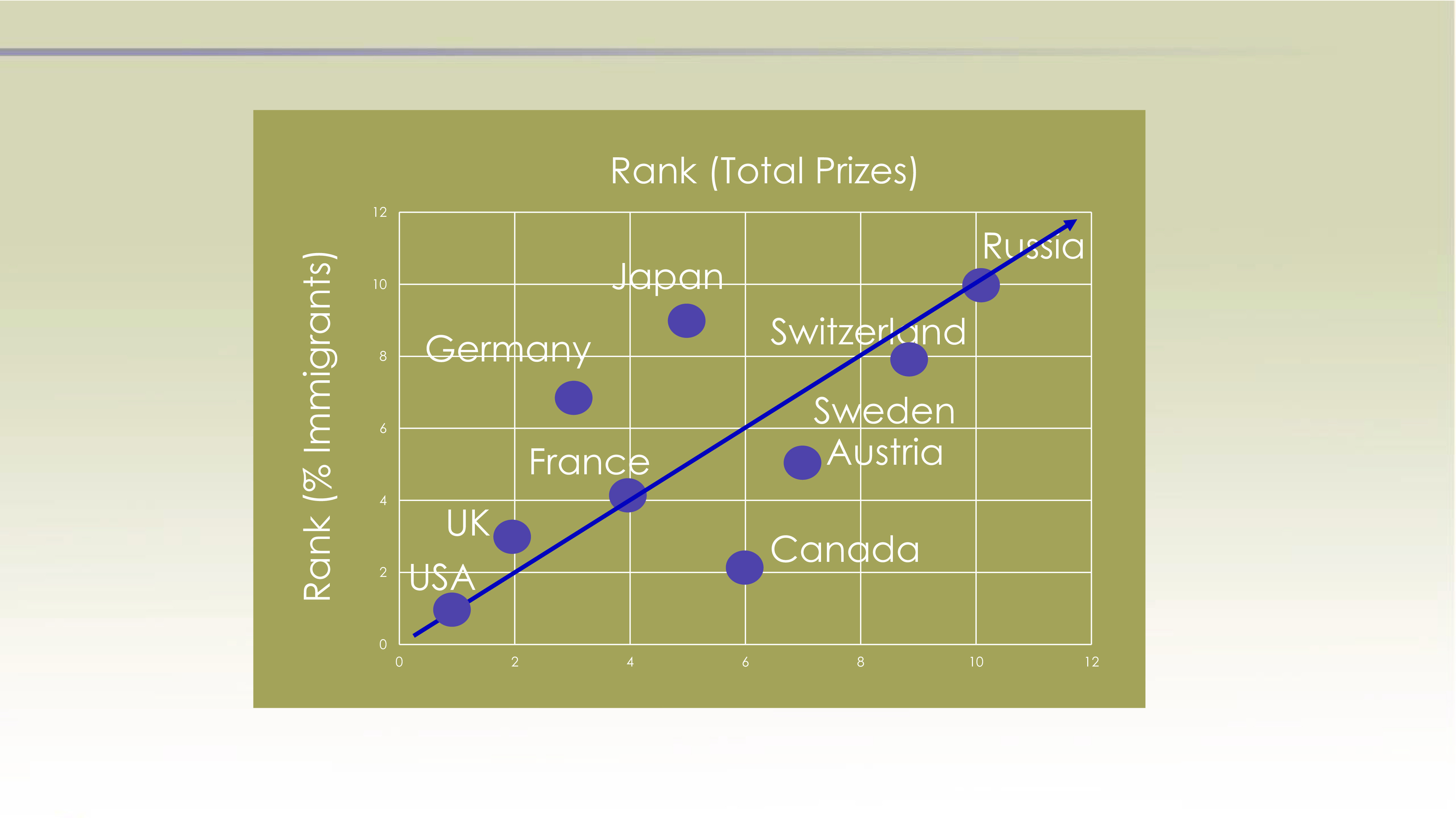
Relationship for the ranking of the top 10 nations in terms of Nobel Prizes plotted against their ranking of the percentage of those prizes that were awarded to first-generation citizens. Sweden and Austria are tied at 7th rank for number of medals and 5th rank for fraction awarded to immigrants. See Dataset S2.

The plot suggests that nations that attract the top science talent globally are rewarded with a larger share of the Nobel prizes in the sciences. Seven of the 10 nations are within one ranking either or both ways of falling on this line. Just two nations, Germany and Japan, plot above the line representing a one-to-one correlation between rankings in Nobel prizes versus percentage of immigrant honorees. Both have lower percentages of immigrants than predicted by a one-to-one correspondence between medal and immigrant rankings. A possible explanation for the success in these two nations despite relatively low representation of immigrants is that Germany and Japan were both restricted from reinvesting in their military after being defeated in WWII. This prohibition allowed both nations to focus their efforts on basic research. Canada has a much higher percentage of foreign-born laureates compared with the nation’s total tally and thus has benefited from science immigration more than any others in the top 10.

With the customary caveat that correlation does not mean causation, I propose the following virtuous cycle that leads to success on the highest podium in science, the Nobel stage. An essential first step is strong funding for basic research. The funding for basic research resulting from Vannevar Bush’s report provided the resources to attract the best and brightest young scholars from the world over who then chose to pursue their careers in America. By attracting such talent, the US research enterprise quickly grew in stature and reputation, resulting in Nobel recognition. Their work also created entirely new industries that spurred growth in the US economy and thus more resources (both public and private) to continue excellence in scientific discovery. This reinvestment in science then continued to lure aspiring researchers to the United States, and the cycle continues. While Nobel prizes might be one of the few internationally recognized measures of research excellence, the impact of immigration on science excellence is apparent in other measures. For example, naturalized citizens represent about 28 percent of the membership of the National Academy of Sciences, identical to 28 percent of US Nobel prizes.

I argue here that maintaining American excellence in science is not entirely about funding, although stagnation in US research funding and huge investments overseas are a major cause for alarm. Having sufficient resources for basic research is essential to support the very best applicants for graduate research assistantships, regardless of nation of origin. But in addition, we must work to keep these talented young scholars here, working in our own R&D enterprise. By the time the United States starts experiencing a noticeable drop in Nobel prizes each year, it will be too late. We will have already lost our edge a decade or more earlier given the current lag time between a major discovery and recognition with a Nobel prize. Concerns are already being voiced that young scientists are finding the United States unwelcoming to foreigners and that they are discovering more attractive opportunities overseas. We must act quickly, for the sake of winning this most noble race, to secure a prosperous future for generations to come.

## Footnotes

^*^More accurately, the Sveriges Riksbank Prize in Economic Sciences in Memory of Alfred Nobel.
